# Phyllodes Tumours Will Only Get Bigger During Pandemic Restrictions

**DOI:** 10.7759/cureus.12313

**Published:** 2020-12-26

**Authors:** Sherif Monib, Hany F Habashy

**Affiliations:** 1 Breast Surgery, West Hertfordshire Hospitals NHS Trsut, St. Albans and Watford General Hospitals, London, GBR; 2 Surgical Oncology, Faculty of Medicine, Fayoum University, Fayoum, EGY

**Keywords:** phyllodes tumour, who classification, mastectomy, adjuvant treatment

## Abstract

Phyllodes tumours of the breast are rare fibroepithelial stromal tumours which are morphologically very different from epithelial breast cancer. Its diagnosis and management has always been challenging till the World Health Organization (WHO) divided it into two-three subtypes in 2003, only then it was found that incidence and management and follow-up of these three subtypes need to be completely different to reach the optimum outcome.

We are presenting a case of a 47-year-old female who presented relatively late (due to pandemic restrictions) with a large phyllodes tumour requiring mastectomy as well as adjuvant treatment.

## Introduction

Phyllodes tumour was first described by Johannes Müller in 1838, as cystosarcoma phyllodes [1], which was later found to be not a very accurate description, as phyllodes tumours are rarely cystic, and the majority of them tend to be more benign than sarcomatous. More than 60 synonyms have been reported for the same condition [2], till the World Health Organisation (WHO) regarded phyllodes tumour as the most appropriate nomenclature [3].

Phyllodes tumours are fibroepithelial tumours that comprise 2-3% of all fibroepithelial breast tumours and 0.3-1.0% of all breast tumours [4,5]. Phyllodes tumours predominantly occur in middle-aged women with average age at presentation, between 40 to 50 years [6], and the average annual incidence of malignant phyllodes tumours is 2.1 per 1 million women [7].

The wide-ranging clinical presentation and pathological features of phyllodes tumours contribute to preoperative diagnostic difficulties. Triple assessment in the form of clinical examination, mammography and/or breast ultrasound scan followed by guided core biopsy is crucial for early diagnosis, classification as well as surgical planning [8].

## Case presentation

We are presenting a case of a 47-year-old female who presented to our outpatient department with left breast large mass which has been steadily growing over the last year, but the patient was a bit apprehensive about seeking medical advice due to the current pandemic restrictions.

**Figure 1:**
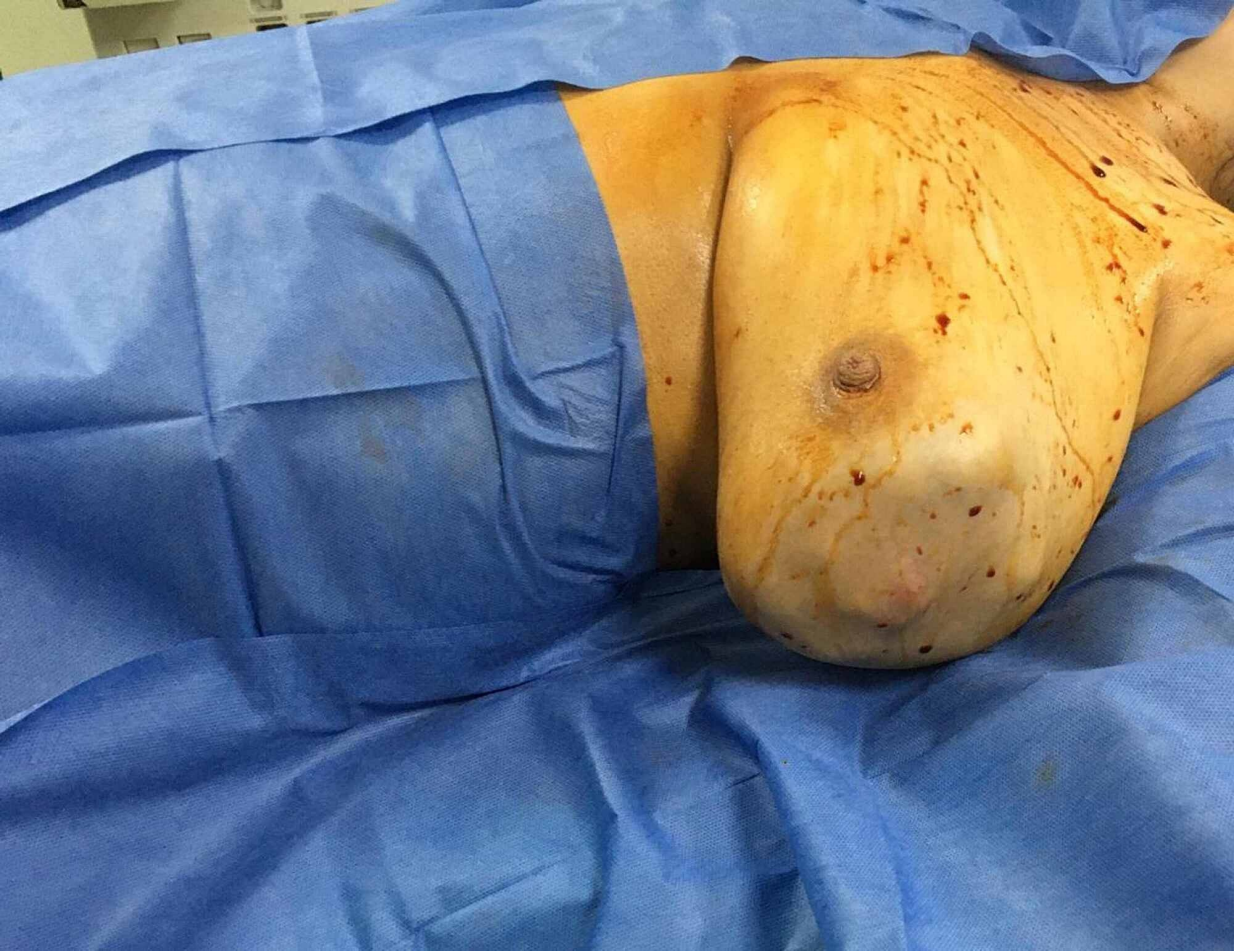
Intraoperative picture showing the large phyllodes tumour occupying the lateral aspect of the breast even seen by inspection.

Her past medical history, and family history, were irrelevant. Breast examination revealed a left breast P4 20mm mass occupying the lateral half of the left breast (Figure 1), with no other palpable ipsilateral or contralateral other breast lumps, axillary or supraclavicular lymph nodes.

**Figure 2:**
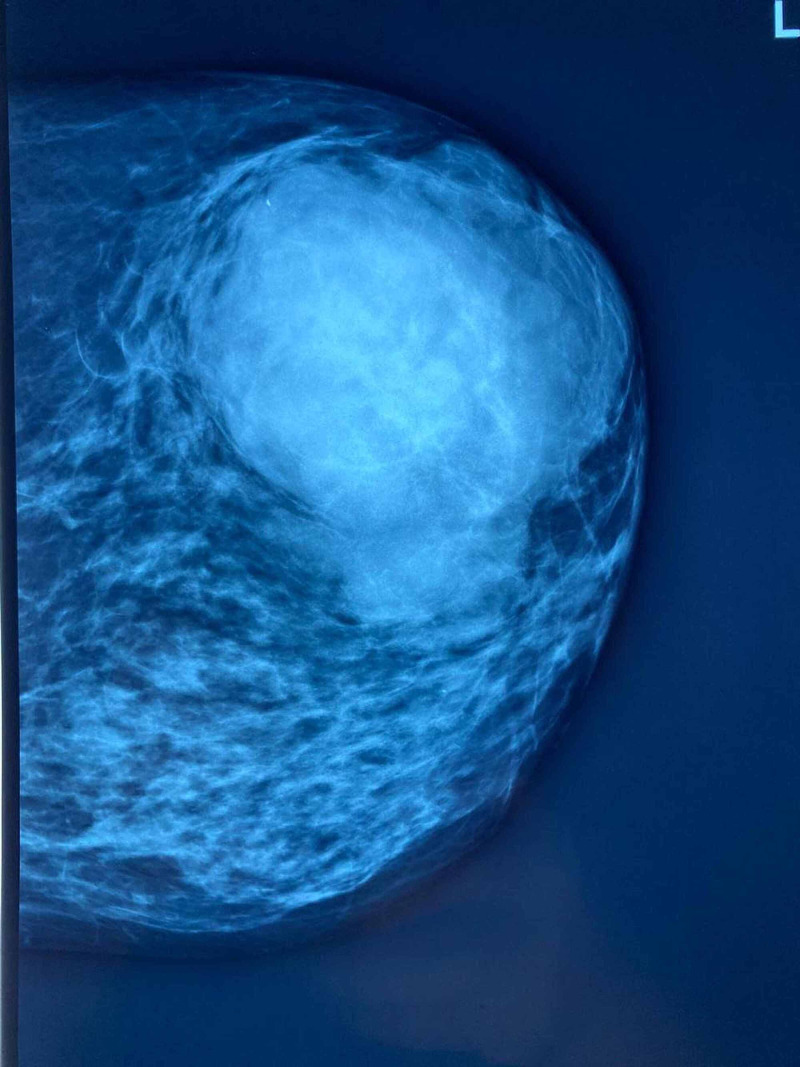
Left craniocaudal mammogram showing the large phyllodes tumour occupying the lateral aspect of the breast.

**Figure 3:**
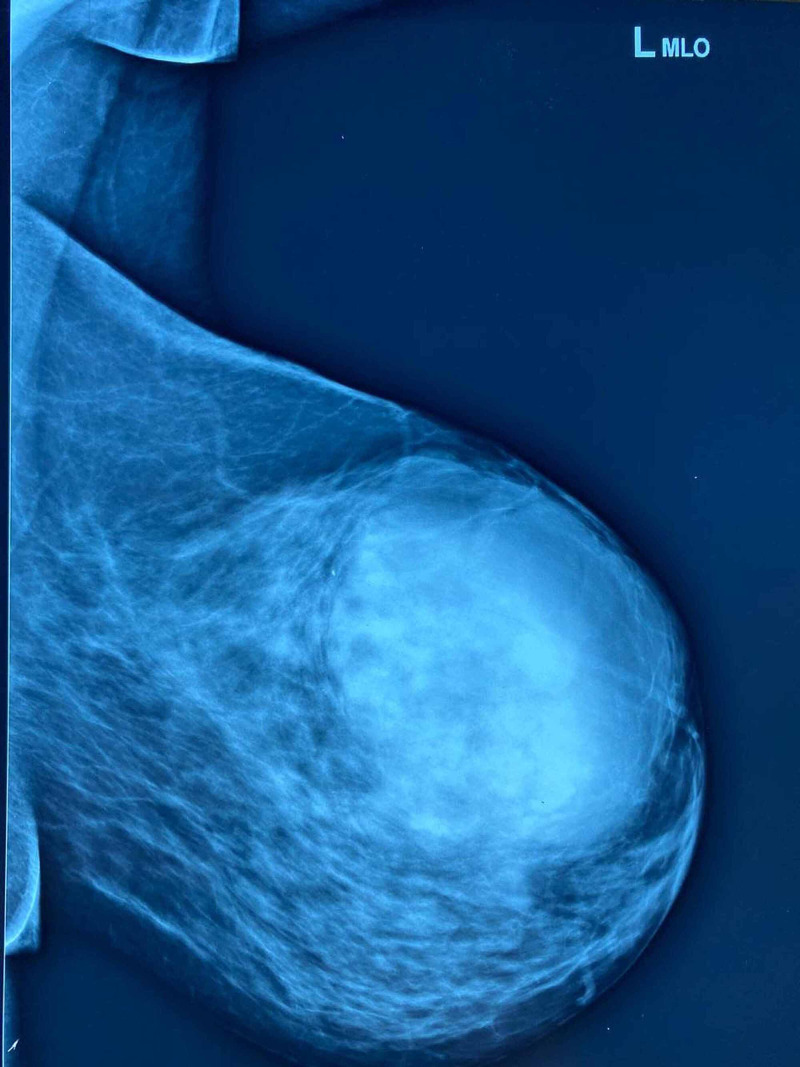
Left mediolateral mammogram showing the large phyllodes tumour occupying the lateral aspect of the breast.

Mammography showed a (Breast Imaging Reporting and Database System) BI-RADS 5 18-mm mass (Figures 2, 3), ultrasound scan-guided core biopsy revealed high-grade malignant phyllodes tumour, and axillary lymph nodes fine needle aspiration cytology (FNAC) showed metastatic disease.

Taking into consideration adequate margins required for complete resection of malignant phyllodes tumours, a mastectomy was carried out. The patient had an uneventful recovery, and she was discharged on the following day, seen in the clinic one week postoperatively, with no postoperative complications noted, and final histology confirmed clear resection margins. Based on the size of the tumour and the likelihood of recurrence, adjuvant chemotherapy, as well as chest wall radiotherapy, was advised. We also arranged for six-monthly chest X-ray and clinical examination, as well as annual right-sided mammogram.

## Discussion

Phyllodes tumours are rare breast tumours which are histologically different from breast carcinoma as they originate from the stromal tissue rather than ductal or lobular tissue [9]. While the majority of these tumours develop de novo, there have been reports of progression of fibroadenoma to phyllodes tumour [10].

Phyllodes tumours can pose a significant burden not only for patients but also for health facilities, as in many cases, diagnosis and management is not as straight forward as expected, with some tumours found to be malignant after excision requiring further re-excisions, adjuvant treatment and lengthy costly follow-up.

Based on histologic features, including stromal cellularity, nuclear atypia, mitotic activity, tumour margin appearance, and stromal overgrowth, the WHO international histological classification group divided phyllodes tumours into three subtypes: benign tumours which represent 58%, borderline tumours which represent 12% and malignant tumours which represent 30% of all phyllodes tumours [4,11].

With surgery, the mainstay of treatment for phyllodes tumours surgical margins ≥10 mm is always recommended [12]; unfortunately, as our patient presented late with a sizable tumour progressively increasing in size. Taking into consideration breast to tumour ratio as well as adequate margins required, we thought that a mastectomy would be a safer option rather than wide local excision. Axillary lymph node clearance is not necessary in all cases and is only indicated in the evidence of lymph node metastasis [13], which was also the case with our patient.

While local recurrence rate is 20%, distant metastases to lungs, bones, brain, and liver is estimated to be around 3.5%; therefore, chemotherapy (ifosfamide, etoposide, doxorubicin, or cisplatin), and radiotherapy should be considered in case of high recurrence or metastatic risk [14,15]. Based on these findings, we have recommended adjuvant chemotherapy and chest wall radiotherapy to our patient.

Since the five-year disease-free survival rate in patients with benign phyllodes tumours is around 96.9%, borderline tumours 83.3%, and malignant tumours 71.7% [16], so every effort should be made to diagnose and treat these patients as early as possible. Early diagnosis of smaller-sized tumours will not only facilitate conservative breast surgery and decrease the need for mastectomy, but also improve prognosis and patient satisfaction.

## Conclusions

In our case, delay in diagnosis and treatment due to the pandemic restriction led to the need for a mastectomy rather than conservative breast surgery. We believe that diagnosis of phyllodes tumours will facilitate a more localised approach of treatment and decrease the need for more radical treatments.

Since phyllodes tumours are morphologically different from epithelial breast cancers, we recommend a multicentric study including large numbers of the three subtypes of phyllodes tumour to be able to come up with solid guidelines for indications and extent of breast and axillary surgery as well as adjuvant treatment and follow up of each subtype to achieve the best outcomes.
